# mRNA-Based Vaccines

**DOI:** 10.3390/vaccines9040390

**Published:** 2021-04-15

**Authors:** Frank Kowalzik, Daniel Schreiner, Christian Jensen, Daniel Teschner, Stephan Gehring, Fred Zepp

**Affiliations:** 1 Pediatric Department, University Medical Center of the Johannes Gutenberg-University, 55128 Mainz, Germany; daniel.schreiner@unimedizin-mainz.de (D.S.); christian.jensen@unimedizin-mainz.de (C.J.); Stephan.Gehring@unimedizin-mainz.de (S.G.); fred.zepp@unimedizin-mainz.de (F.Z.); 2 Department of Hematology, Medical Oncology, and Pneumology, University Medical Center of the Johannes Gutenberg University, 55122 Mainz, Germany; daniel.teschner@unimedizin-mainz.de

**Keywords:** mRNA, vaccine constructs, delivery systems, lipid nanoparticles, cancer, infectious disease

## Abstract

Increases in the world’s population and population density promote the spread of emerging pathogens. Vaccines are the most cost-effective means of preventing this spread. Traditional methods used to identify and produce new vaccines are not adequate, in most instances, to ensure global protection. New technologies are urgently needed to expedite large scale vaccine development. mRNA-based vaccines promise to meet this need. mRNA-based vaccines exhibit a number of potential advantages relative to conventional vaccines, namely they (1) involve neither infectious elements nor a risk of stable integration into the host cell genome; (2) generate humoral and cell-mediated immunity; (3) are well-tolerated by healthy individuals; and (4) are less expensive and produced more rapidly by processes that are readily standardized and scaled-up, improving responsiveness to large emerging outbreaks. Multiple mRNA vaccine platforms have demonstrated efficacy in preventing infectious diseases and treating several types of cancers in humans as well as animal models. This review describes the factors that contribute to maximizing the production of effective mRNA vaccine transcripts and delivery systems, and the clinical applications are discussed in detail.

## 1. Introduction

Increases in the world’s population (approaching 7.8 billion), population density, global travel, and contact between people promotes the spread of emerging pathogens. Zoonosis represents a constant threat to introduce previously uncharacterized pathogens, such as HIV, SARS, MERS CoV, and SARS-CoV-2, into the population [1,2]. Vaccines are the most cost-effective strategy to prevent this global spread and to suppress both acute and chronic infections.

Conventional vaccines are generally classified as live, attenuated, or non-live. Vaccination with attenuated pathogens has successfully decreased the burden of a number of infectious diseases, e.g., smallpox, polio, measles, mumps, and rubella. Conventional vaccines comprised of attenuated viruses such as these take years to develop due to the time required to collect and subsequently adapt (attenuate) the virus in vitro. Adaptation may be hampered by a number of factors including difficulty cultivating the pathogen in specialized, biosafety level facilities. Moreover, in addition to a customized production process, each new conventional vaccine requires complex purification and testing. Notably, in this regard, live attenuated vaccines carry a credible risk of reversion [1].

The antigenic component of non-live vaccines can be the whole inactivated organism, purified proteins derived from the organism (e.g., tetanus or diphtheria toxoid), recombinant proteins such as those that comprise hepatitis B virus and human papillomavirus vaccines, or polysaccharides found in the pneumococcal vaccine for *Streptococcus pneumoniae*. Non-live vaccines are often combined with adjuvants to promote immunogenicity.

Traditionally, vaccine development is a complex expensive, slow, laborious undertaking that requires substantial investment [3]. Creating a new vaccine candidate using established technologies is estimated to cost >500 million USD, with additional expenses of 50 to 700 million USD required to retrofit manufacturing facilities and equipment [4]. Indeed, the need for dedicated production processes and facilities for each vaccine created using conventional technologies keeps validation and manufacturing costs high [1]. Furthermore, the average development of a conventional vaccine from the preclinical phase requires >10 years and has a market entry probability of 6%. The long lead time and hundreds of potentially complex steps required for manufacturing highlight the urgent need for new approaches to expedite vaccine development.

Established methods used to identify and produce new vaccine candidates are no longer sufficient to ensure global protection against emerging, often ill-defined pathogens; a shift in methodology is needed. Manufacturers desperately require new technologies able to spur rapid vaccine development and large scale production, reduce the cost, shorten the time to licensure, and to allow responding quickly to pandemic threats [1]. Viral vector and nucleic acid-based vaccine platforms created during the past few decades promise to provide solutions to these vaccine challenges [1,5]. Viral vector vaccines are comprised of nonreplicating or attenuated, replicating recombinant viruses that encode one or more target antigens. Presentation of these antigens in combination with stimuli inherent in the vector mimic a natural infection that induces strong humoral and cell-mediated immunity. A potential disadvantage of viral vector vaccines is pre-existing immunity to the vector, such as a recombinant adenovirus, which commonly causes human infection.

## 2. mRNA Vaccines

Vaccination with non-viral vector delivered nucleic acid-based vaccines mimics infection or immunization with live microorganisms. mRNA technology promises to dramatically change the traditional approach to vaccine development. The underlying principle is delivery of a transcript that encodes one or more immunogens into the host cell cytoplasm, where translation generates immunogenic proteins that are subsequently sequestered intracellularly, incorporated into the cell membrane, or secreted.

The mRNA is generated by transcribing a DNA template synthesized once the genetic sequence encoding the immunogen is known and disseminated globally. The design and manufacturing of mRNA-based vaccines on a clinical scale is possible within weeks from the time the antigenic sequence becomes available. mRNA production is cell-free, using in vitro transcription methodology. Both the template and transcript can be produced in the laboratory using materials that are readily accessible. Moreover, a facility dedicated to mRNA production should be able to manufacture vaccines quickly against multiple targets with only minimal adaptation.

mRNA vaccines exhibit a myriad of advantages related to safety, efficacy, and production compared to more conventional approaches. Vaccine production involves neither infectious elements nor a risk of stable integration into the host cell genome; rather, the vaccine RNA strand is degraded once the protein is produced. Moreover, vaccination is capable of generating humoral and cell-mediated immunity, which is well-tolerated by healthy individuals with few side effects. Notably, mRNA vaccines induce potent MHC class I-restricted CD8^+^ as well as MHC class II-restricted CD4^+^ T-cell responses. Production and accumulation of the immunogen in the cytoplasm can be processed efficiently, and the epitopes are presented in association with MHC class I molecules on the cell surface. Epitopes derived from secreted or recycled proteins encoded by the vaccine can be presented in association with MHC class II molecules. mRNA is the minimal genetic vector; therefore, anti-vector immunity is precluded, and vaccines can be administered repeatedly. As such, an individual can potentially be immunized with multiple different mRNA vaccine constructs created using the same technology.

Since production is cell-free and laboratory based, mRNA vaccines are less expensive than conventional vaccines and can be produced more rapidly by processes that can be standardized and scaled-up, improving responsiveness to large emerging outbreaks [6]. Moreover, mRNA vaccine constructs can be readily modified in order to eliminate undesired side effects or to enhance immunogenicity, e.g., to respond to mutations and antigenic changes in the organism.

**Figure 1:**
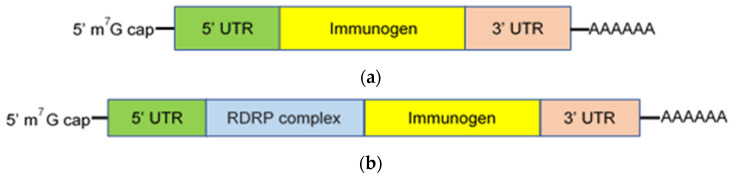
mRNA vaccine constructs. Constructs are classified as either nonreplicating (**a**) or self-replicating (**b**) and composed of a 5′ m^7^G cap, 5′ and 3′ untranslated regions (UTR) which flank the nucleotide sequence that encodes the immunogen of interest, and a 3′-poly(A) tail. Additionally, self-replicating mRNA constructs encode an RNA-dependent RNA polymerase (RDRP) complex that transcribes and amplifies the message.

mRNA based vaccines are generally classified as either conventional, nonreplicating, or self-replicating (self-amplifying). Nonreplicating mRNA constructs are small in size, simple, and lack additional encoded proteins capable of inducing unintentional immune responses [7]. They encode the immunogen of interest, which is flanked by 5′ and 3′ untranslated regions (UTRs), a 5’ cap structure consisting of 7-methylguanosine (m^7^G) connected by a triphosphate bridge to the first nucleotide, and a 3′-poly(A) tail (Figure 1A). The 5′ m^7^G cap blocks recognition by the cytoplasmic RNA sensor, RNA helicases retinoic acid-inducible gene I (RIG-I), suppresses 5′–3′ exonuclease-mediated degradation, recruits translation initiation factors, and promotes efficient translation [8]. The length, structure, and regulatory elements within both the 5′ and 3′ UTR regions also contribute to maximum gene expression [9,10]. The poly(A) tail and its length are critical for translation and protection of the mRNA vaccine construct from degradation [11,12]. Translation efficiency is also enhanced by sequence engineering (codon optimization) and nucleoside modification (e.g., replacement of uridine with pseudouridine), which suppresses Toll-like receptor (TLR) recognition and the innate immune response to mRNA constructs [13,14,15]. mRNA purity is essential; DNA-dependent RNA polymerases yield small oligoribonucleotide as well as double-stranded RNA impurities during construct synthesis [16,17]. Removal of these impurities, which are recognized by pattern recognition receptors, promotes translation and protein synthesis by suppressing the innate immune response and the production of type I interferon and inflammatory cytokines [18].

Self-replicating mRNA constructs (replicons) encode an RNA-dependent RNA polymerase (RDRP) complex required for self-amplification as well as the components found in nonreplicating constructs (Figure 1B) [19,20]. The RDRP complex is often derived from alphaviruses, e.g., Sindbis virus [19,20]. Self-replication increases the magnitude and duration of construct expression and, consequently, production of the encoded immunogen. In non-human primates (NHP), low doses of self-replicating mRNA vaccine induced enhanced immunogen production for an extended duration, where production peaked on day 3 and remained detectable for more than 14 days following immunization [20]. Similarly, immunization with a self-replicating mRNA construct induced more protein synthesis for a longer period of time and a greater immune response in mice, compared with a nonreplicating mRNA vaccine [21]. An additional advantage of self-replicating mRNA constructs is the ability to incorporate multiple gene sequences into the same replicon, allowing the expression of both the target immunogen and immunomodulatory molecules such as CD40L, CD70, OX40L, and GM-CSF to enhance potency [22,23].

Self-replicating constructs are much larger than nonreplicating mRNA constructs (i.e., 9.3 versus 2.2 kb), making production and stability more challenging and possibly limiting vaccine internalization [23,24]. They tolerate few nucleotide modifications or sequence alterations without losing self-amplifying activity [23]. Moreover, self-replicating constructs include unrelated antigenic proteins (i.e., the RDRP complex) capable of inducing a strong immune response that suppresses translation and immunogen production. Furthermore, double-stranded RNA intermediates formed during self-replication are the natural ligands of cytoplasmic RNA sensors RIG-I and melanoma differentiation-associated protein 5 (MDA5). Ligation by these receptors initiates the release of type I interferons (INFα/β) and activation of the interferon response gene cascade and innate immunity [25]. In fact, it has been suggested that the strong intrinsic adjuvant activity of self-replicating mRNA contributes to its higher immunogenicity at lower doses compared to nonreplicating mRNA constructs [20,22]. Notably, whether a type I INFα/β response is beneficial or detrimental to the generation of vaccine-induced immunity is a matter of ongoing discussion [26,27]. Consequently, IFNα/β production should be considered in the design of any mRNA vaccine given evidence to support the ability of IFNα/β to both enhance and inhibit protective immunity [6].

While conventional, nonreplicating mRNA vaccine constructs effectively elicit immune responses, they are constrained by relatively short half-lives. Self-replicating mRNA vaccine constructs exhibit comparative increases in the magnitude and duration of message expression, and immunogen production [24,28]. However, their size makes production and stability more challenging. Circularization of exogenous mRNA offers a potential means of extending message expression and immunogen production, and a promising alternative to self-replicating mRNA vaccines.

Circular RNAs (circRNAs) are a class of single-stranded RNAs with a covalently closed loop structure [29]. They are endogenous in eukaryotic cells and conserved across species. Most are created from linear mRNA sequences by back-splicing; the vast majority appear to fulfill noncoding roles that are not completely understood. circRNAs lack end motifs necessary for the interaction with a number of cellular proteins and, as such, are resistant to exonuclease-mediated degradation. Recently, Wesselhoeft and collaborators reported a technology for circularizing a wide range of mRNAs based upon self-splicing by an autocatalytic intron, which stabilizes and extends the half-lives of messages and prolongs production of encoded proteins [30,31]. An integrated coxsackievirus B3 internal ribosome entry site facilitates ribosomal binding and circRNA translation. Exogenous circRNAs avoid recognition by cellular RNA sensors (i.e., RIG-I) and TLR without nucleoside modification, thereby abrogating innate immune responses in animal models as well as cultured TLR-expressing cells. These finding suggest that circRNA vaccine constructs could provide an alternate, improved approach to RNA-based vaccination. Indeed, the potential use of circRNA-based vaccines in cancer stem cell therapy has been proposed [32].

## 3. Delivery Systems

Chemical modifications and sequence engineering have improved both the translation and shelf life of synthetic mRNA vaccines [24]. Naked mRNA, however, is unsuitable for therapeutic use. A key factor inhibiting mRNA vaccine development, until recently, was the absence of an efficient, well-tolerated delivery system. The biggest barrier lies in the need for cellular uptake and translocation. The negative potential across the cell membrane creates a formidable barrier for mRNA molecules. Naked mRNA, prone to nuclease digestion, is too large and highly negatively charged to passively cross the cell membrane [33]. Purportedly, the rate of naked mRNA uptake by cells is less than 1 in 10,000 molecules [34]. Relatively little internalized naked mRNA is translated but, instead, rapidly degraded. Moreover, naked mRNA injected directly into humans or animals can elicit severe inflammatory, innate immune responses independent of the protein encoded.

Electroporation with a gene gun offers means of vaccinating with naked mRNA [35]. mRNA molecules pass through membrane pores formed by a high-voltage pulse directly into the cell cytoplasm. Though electroporation is an efficient means of mRNA delivery and vaccination in mouse models, there is no conclusive data to support the efficacy of this approach in humans or large animals.

Dendritic cells (DCs) are professional antigen-presenting cells that play a key role in initiating immune responses. DCs are highly amenable to mRNA transfection ex vivo and, thus, provide another means of vaccinating with naked mRNA. Vaccines consisting of mRNA-transfected DCs elicit cell-mediated immune responses primarily and, therefore, are principally used in cancer immunotherapy [36]. Indeed, DCs transfected with mRNA ex vivo for adoptive transfer to cancer patients was the first mRNA-based vaccine to enter clinical trials [37]. Reinfusing recipients with autologous DCs transfected with antigen-encoding mRNA ex vivo, however, is very expensive and labor intensive. Moreover, while clinical trials demonstrated that the infusion of mRNA transfected DCs to be safe and well tolerated, with an immune response detectable in more than 50% of vaccinated patients, clinical responses were sporadic or very limited [38]. Thus, infusion with transfected DCs offers only a short-term approach to cancer treatment. For the long term, the targeting of antigen-encoding mRNA to specific DC subsets in vivo is envisioned [38,39,40].

**Figure 2:**
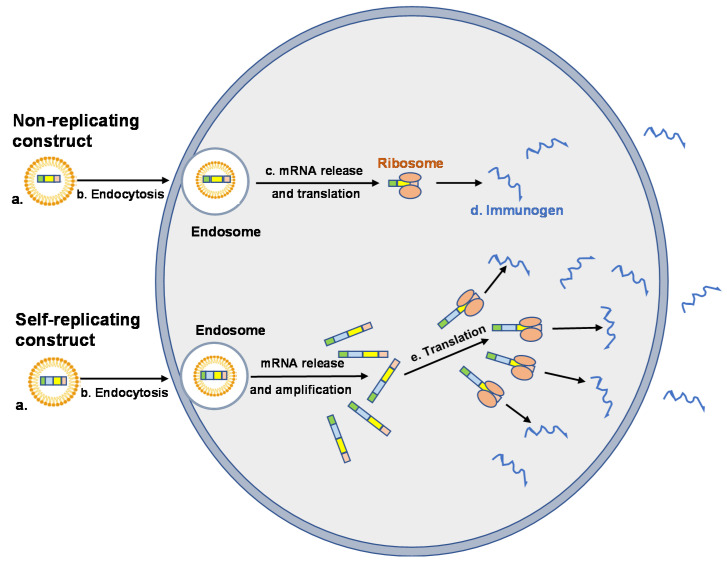
Cellular uptake and expression of vehicle encapsulated mRNA vaccine constructs. Nonreplicating and self-replicating vaccine constructs (NRC and SRC, respectively) are encapsulated in LNP to prevent degradation and to promote cellular uptake (**a**). Uptake of the mRNA–LNP complex is mediated by endocytosis (**b**). mRNA vaccine constructs are released from the endosome into the cytosol where NRC are translated by ribosomes (**c**) and the immunogen produced (**d**). SRC are translated, producing the RNA-dependent RNA polymerase (RDRP) necessary for self-amplification and production of the immunogen (**e**). Immunogens (sequestered intracellularly, incorporated into cell membranes or secreted) induce humoral and cell-mediated immune responses.

The inflammatory profile of synthetic mRNA can be altered significantly by a number of factors that includes nucleoside modification, sequence engineering, purification, and incorporation into a delivery vehicle [6,39]. The primary functions of the vehicle are to protect the message from extracellular nuclease digestion and to facilitate uptake by host cells, primarily by endocytosis followed by electrostatic attachment and fusion with the cell membrane [41] (Figure 2). Once internalized, the delivery vehicle must promote endosomal escape and the release of its contents into the cytosol, where translation occurs. While internalization is a relatively simple process, endosomes and the endosomal membrane represent a significant barrier to the release and expression of intact mRNA [42].

**Figure 3:**
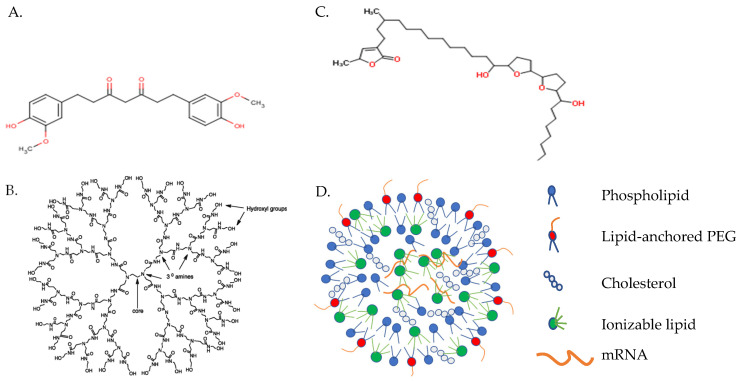
mRNA-based vaccine carriers. (**A**) Diethylaminoethyl-dextran; (**B**) cationic polyamidoamine dendrimer; and (**C**) protamine. Images captured from PubChem (https://pubchem.ncbi.nlm.nih.gov, accessed on 8 April 2021) (**D**) Lipid nanoparticle.

A number of polymer, peptide, and lipid-based carriers have demonstrated transport efficacy in preclinical and some clinical trials [6]. Diethylaminoethyl dextran (Figure 3A), the first polymer tested as a delivery reagent for in vitro transcribed mRNA, was 100 to 1000 times less efficient, however, than lipid-mediated transfection [43]. Alternatively, cationic dendrimers (Figure 3B) have been widely studied for gene delivery but only used for mRNA-based vaccine delivery in a few studies. Cationic dendrimers are highly branched, polymeric macromolecules which are symmetric around a core, often adopting a spherical three-dimensional structure that allows them to pass freely through cell membranes, unlike classical polymers [41]. Though widely examined as a method for DNA delivery, only a few studies have explored cationic dendrimers as an mRNA-based vaccine delivery system. Chahal and coworkers, for example, demonstrated the capacity of dendrimer-based nanoparticles condensed around a self-replicating mRNA vaccine construct to induce protective immunity against Ebola, influenza, and Zika viruses in mice administered a single dose intramuscularly (i.m.) [44,45]. Future use of dendrimers as vaccine carriers may be limited, however, by steric factors that inhibit dendrimer biodegradation and, thus, enhance its accumulation in the tissues and toxicity.

Recently, Rauch et al. reported the efficacy of vaccine constructs composed of mRNA complexed with a small, arginine-rich nucleotide-binding peptide, protamine (Figure 3C) [46]. Cell-penetrating peptides such as protamine are capable of binding, stabilizing, and transporting mRNA into the cytoplasm [47]. The RNActive vaccine platform (CureVac AG; Tübingen, Germany) composed of mRNA (customized to maximize the level and duration of immunogen production) complexed with protamine demonstrated potency against cancer and a variety of infectious diseases in animal models [1,47,48]. The results achieved by vaccination with mRNA encoding rabies virus glycoprotein (RABV-G) complexed with protamine in a phase 1 clinical trial were suboptimal, though were much improved in preclinical studies by adopting a lipid-containing nanoparticle (LNP) delivery system [49]. A phase 1 clinical trial involving mRNA encoding RABV-G complexed with LNP is currently in progress (ClinicalTrials.gov Identifier: NCT03713086).

Vectors based upon lipids or lipid-like compounds are, by far, the most common non-viral gene carriers [23]. Recent studies have focused upon the development of novel ionizable lipids and formulations that improve cellular uptake, endosomal release, and mRNA expression [28,42]. Co-formulation into ionizable LNPs was first developed for siRNA delivery [50]. LNPs are typically synthesized by mixing mRNA in an acidic aqueous phase with an ethanol phase containing precise molar ratios of (1) an ionizable cationic lipid (6–7 pKa value in the LNP) that encapsulates polyanionic mRNA at low pH; (2) a zwitterionic lipid that resembles the lipids in cell membranes; (3) cholesterol, which stabilizes the LNP lipid bilayer and promotes fusion; and (4) lipid-anchored polyethylene glycol to reduce non-specific protein absorption, diminish NPC aggregation, and improve colloidal stability (Figure 3D) [23,50]. LNPs generally contain mRNA at a relatively low copy number (1–10), which is bound by ionizable cationic lipid and located in the particle core [51].

Multiple reports indicate that ionizable lipid is the principal factor determining the efficacy of LNPs designed for siRNA delivery [50]. It is neutral at physiological pH, eliminating any cationic charge in the circulation, but becomes protonated at pH ~6.5 in the endosome, facilitating endosomal release [51]. Specific LNP formulations are often proprietary. In this regard, Hassett et al. evaluated the ability of a group of proprietary biodegradable ionizable lipids incorporated into LNPs to maximize the expression and immunogenicity of encapsulated mRNA [52]. A formulation was identified that produced a vigorous immune response with enhanced safety relative to other formulations in both mice and NHP primates inoculated i.m. Safety and tolerability, key factors in the performance of any new vaccine, were improved by the inclusion of biodegradable lipids with short half-lives in the LNPs. Increased biodegradability correlates with a reduction in inflammation at the injection site.

It is essential that the components of LNPs are susceptible to rapid metabolism or excretion to avoid accumulating in tissues and attending adverse consequences [39]. Notably, in addition to facilitating uptake by cells and gene expression, delivery systems can stimulate innate immunity and, consequently, provide an adjuvant effect [53,54]. LNP formulations that induce potent immune responses, despite a reduction in inflammatory cell infiltration and cytokine production, however, support the conjecture that mRNA vaccines do not require strong adjuvant activity [52]. LNPs offer the additional advantage that they can be formulated to encapsulate multiple mRNAs encoding different proteins and immunostimulants (e.g., pembrolizumab, anti-PD-1 receptor) into a single vaccine to improve overall immunogenic activity (for examples, see ClinicalTrials.gov Identifiers: NCT03313778, NCT03897881, and NCT04232280).

Targeting antigen-presenting cells, i.e., DCs, is a principal goal of any vaccine delivery system. Targeted delivery reduces the required mRNA dosage and any potential off-target side effects. In this regard, Perche et al. demonstrated that transfection of mannose-receptor-expressing dendritic cells was increased when mice were inoculated with mRNA incorporated into mannosylated lipid particles [55]. Additional efforts to target mRNA delivery to DCs have focused upon functionalizing nanoparticles with monoclonal antibodies that recognize and bind cell surface DC receptors such as DC-SIGN, DEC-205, and langerin [56,57,58]. Use of targeting moieties, though, usually does not prevent the uptake of mRNA–LNP constructs by the liver and spleen. Moreover, this approach has only limited value: (1) chemical conjugation of monoclonal antibody to the LNP surface is inefficient, (2) serum proteins adsorb to the surface of LNPs and often form a corona that masks the specificity of the attached antibody and, finally, (3) antibody bound to the targeted receptor could possibly trigger signaling pathways that lead to unwanted consequences.

Alternatively, DCs in the lymphoid compartment can be specifically and effectively targeted without functionalization by adjusting the negative net charge of the LNP formulation [40]. Particles with a slight negative charge (i.e., 1.3:2, positive to negative) effectively targeted mRNA to the spleens of mice injected intravenously. mRNA uptake was highest in splenic macrophages, but the highest transfection and translation rates occurred in conventional DCs. Moreover, vaccination induced strong effector and memory T-cell responses and the rejection of tumors in a mouse model. In a similar vein, rhesus macaques immunized i.m. or intradermally (i.d.) with an LNP-encapsulated mRNA vaccine construct that encoded influenza H10 hemagglutinin exhibited rapid mobilization of professional antigen-presenting cells (APCs), i.e., monocytes and DCs, to the site of administration [59]. These cells quickly engulfed the mRNA construct, translated the message, and upregulated key co-stimulatory receptors, e.g., CD80 and CD86. Injected LNP less than 150 nm in size readily entered the lymphatic vessels from interstitial spaces, enabling both passive and APC-associated vaccine transport to the draining lymph nodes [58,59,60]. The lymph nodes provide a rich environment for naïve T-cell priming by APCs expressing the product(s) of mRNA translation. Consequently, the vaccinated macaques exhibited H10-specific CD4^+^ and CD8^+^ T-cell responses and protective anti-HA titers [59].

mRNA vaccines can be administered by needle or needle-free methods; the route of administration plays an important role in determining vaccine efficacy [61]. Efficacy and safety are influenced by the anatomical and physiological properties of the vaccination site. While the optimal route for mRNA vaccine delivery remains to be determined, i.m. injection is, currently, the most commonly used route [23,61]. Muscle contains a large network of blood vessels which can recruit and recirculate APCs to draining lymph nodes. The injection volume is large (1–3 mL adults), invasion is minimal, and local side effects are limited.

Intradermal vaccination offers a reasonable alternative to i.m. injection. In this regard, macaques vaccinated i.d. with mRNA–LNP, in the study cited above, exhibited significantly higher anti-HA titers and a greater number of H10-specific CD4^+^ T cells than did the i.m. vaccinated group [59]. Furthermore, additional studies suggest that i.d. vaccination may require a much smaller dose than that administered i.m. to elicit a comparable immune response, an important consideration when the vaccine supply is limited [58].

## 4. Clinical Applications

### 4.1. Cancer

The majority of early work with mRNA vaccines has focused on cancer. Clearly, conventional vaccine approaches are not applicable to such non-infectious diseases. Cancer vaccines are therapeutic, rather than prophylactic, designed to target tumor-associated antigens expressed preferentially by cancerous cells and, as a result, to stimulate cell-mediated immune responses capable of reducing the tumor burden. Exploration of mRNA to induce adaptive immune responses to cancer began in 1995, when Conry and coworkers reported that protective antitumor immunity could be induced in mice by intramuscular injection of mRNA encoding carcinoembryonic antigens [62]. Currently, more than 100 clinical trials for mRNA vaccines are listed by the U.S. National Library of Medicine (ClinicalTrials.gov) for a wide range of cancers that includes breast, ovarian, prostate, colon, metastatic renal cell, glioblastoma, melanoma, and solid tumors. Most of these trials are early, but some have progressed to phase 2.

The most straightforward approach using mRNA to vaccinate against cancer is to immunize patients with vaccines that encode tumor-associated antigens (TAAs). This approach is typified by RNActive^®^ technology (CureVac AG) in which mRNA, customized using proprietary methods to maximize protein synthesis, is complexed with protamine to promote Th_1_-type T-cell responses [46,47,63]. The resultant vaccine constructs induced both humoral and cell-mediated responses in animal models, and are currently being evaluated in several clinical trials. In one phase 1/phase 2 trial (ClinicalTrials.gov Identifier: NCT00831467), for example, hormonal refractory prostate cancer patients were intradermally treated with a vaccine construct that encodes four prostate-specific antigens: PSA, PSMA, PSCA, and STEAP [63]. No results have been reported to date.

Personalized mRNA vaccine constructs offer a second approach to immunizing cancer patients [64]. Somatic mutations are important drivers of cancer development. Many mutations are unique, leading to a distinct set of mutations in each patient’s tumor (the mutanome), defined by comparing exome sequencing data obtained by next generation sequencing of healthy and tumor-derived tissues. Evidence suggests that a significant subset of these mutations encode neoepitopes recognized by autologous T cells [37]. These epitopes are evaluated to determine those that are not subject to central immune tolerance and, therefore, confer antitumor vaccine activity. Given the flexibility and ease of manufacturing, mRNA sequences encoding multiple neoepitopes can be incorporated into a single, poly-neoepitope backbone that comprises the personalized vaccine construct. The safety and clinical feasibility of this approach was demonstrated in a first-in-human trial undertaken to treat 13 patients with metastatic melanoma (ClinicalTrials.gov Identifier: NCT02035956) [65]. Each patient was immunized with a vaccine that encoded 10 neoepitopes that were unique to his/her tumor. All vaccinated patients exhibited CD4^+^ and CD8^+^ T-cell responses to selected epitopes. Antitumor responses were detected in some patients in whom vaccine-induced T-cell infiltration and neoepitope-specific killing of autologous tumor cells were found in metastases resected post-vaccination. Since this initial report, therapeutic cancer treatment with personalized mRNA vaccines has received significant attention; several clinical trials listed by the U.S. National Library of Medicine are currently ongoing (see ClinicalTrials.gov).

Notably, cancer vaccine trials involving mRNA vaccine constructs have not been very successful in treating late-stage patients with treatment-refractory tumors [66,67]. Consequently, in an ongoing phase 2 study (ClinicalTrials.gov Identifier: NCT03897881), for example, patients with high-risk melanoma are being treated with personalized cancer vaccine mRNA-4157 with or without pembrolizumab, a monoclonal antibody that binds the PD-1 receptor and blocks the interaction of PD-L1 and PD-L2, thus restoring T-cell activity. Similarly, investigators propose treating patients who have resected or unresected solid tumors with personalized mRNA-4157 vaccine in combination with pembrolizumab (ClinicalTrials.gov Identifier: NCT03313778).

### 4.2. Infectious Diseases

Conventional vaccines are largely prophylactic, created to prevent infectious diseases. Traditional approaches to develop new vaccines are challenged by requirements for rapid development and large scale implementation [24]. In this regard, a number of recent reports demonstrated the potency and versatility of mRNA vaccine constructs to elicit protection against a wide variety of infectious agents (e.g., Zika virus, rabies, influenza virus, cytomegalovirus, Ebola virus, *Streptococcus* species, and *Toxoplasma gondii*) in animal models. mRNA-based vaccines constructs demonstrated the ability to generate potent neutralizing antibody responses in animals immunized with only one or two low doses.

Small animals and NHPs immunized with an mRNA–LNP vaccine construct that encoded the pre-membrane and envelope (prM-E) glycoproteins of Zika virus, for example, exhibited a strong, durable neutralizing antibody response that conferred sterilizing immunity to ZIKV infection [68,69]. Similarly, NHPs vaccinated i.m. with an LNP-formulated mRNA vaccine that encoded the rabies virus glycoprotein (RABV-G) produced protective antibody titers that could be boosted and remained stable for 1 year [70]. The same study also reported that NHPs immunized with a single dose of an mRNA–LNP vaccine construct that encoded the hemagglutinin glycoprotein of the H1N1pdm09 influenza virus strain induced anti-H1N1-HI titers that were equal or greater than those considered protective in humans and equivalent to those elicited by a licensed inactivated influenza virus vaccine Fluad.

The results of animal studies such as these has generated a great deal of enthusiasm. mRNA vaccines are currently being tested clinically for a number of viral diseases, including rabies virus, influenza virus, Zika virus, cytomegalovirus, respiratory syncytial virus, and novel coronavirus (SARS-CoV-2). With the exception of SARS-CoV-2, however, none of these clinical trials has passed the early phase. In this regard, Alberer et al. reported that CV7201 (an mRNA vaccine construct that encodes RABV-G complexed with protamine) was reactogenic and elicited acceptable virus neutralizing antibody titers in only 71% and 46% of study participants inoculated i.d. and i.m., respectively (ClinicalTrials.gov Identifier: NCT02241135) [71]. In a more recent study (ClinicalTrials.gov Identifier: NCT03713086), however, 1 or 2 small doses of CV7202 (mRNA encoding RABV-G in a LNP complex) administered i.m. elicited rabies virus neutralizing antibody titer ≥0.5 IU/mL, an acceptable value by WHO standards [72]. Feldman et al. reported similar findings [73]. H10N8 and H7N9 mRNA influenza vaccine constructs encoding the hemagglutinin of the H10N8 and H7N9 influenza strains formulated in an LNP delivery system induced hemagglutination inhibition titers >1:40 and microneutralization titers >1:20 in ≥90% of the study volunteers inoculated i.m., albeit that a significant T-cell response to vaccination was not found (ClinicalTrials.gov Identifiers: NCT03076385 and NCT03345043).

SARS-CoV-2 and the coronavirus disease 2019 (COVID-19) pandemic demonstrate the urgent need for technologies that are flexible and able to achieve rapid vaccine development and large scale production. Three of 52 vaccine candidates currently undergoing clinical evaluation (WHO draft landscape of COVID-19 candidate vaccines, 10 December 2020) are mRNA-based vaccines involved in phase 2b/3 (CureVac, ClinicalTrials.gov Identifier: NCT04515147) or phase 3 (Pfizer-BioNTech, ClinicalTrials.gov Identifier: NCT04368728 and Moderna/NIAID, ClinicalTrials.gov Identifier: NCT04470427) trials. Two—mRNA-1273 vaccine (Moderna TX) and BNT162b2 vaccine (Pfizer-BioNTech)—received emergency use authorization from the U.S. Food and Drug Administration and are currently being administered prophylactically to prevent COVID-19.

The BNT162b2 vaccine produced by Pfizer-BioNTech is an LNP-formulated, nucleoside-modified RNA vaccine that encodes a prefusion stabilized, membrane-anchored SARS-CoV-2 full-length spike protein [74,75]. BNT162b2 vaccination elicits high SARS-CoV-2 neutralizing antibody titers and robust antigen-specific Th_1_-type CD4^+^ and CD8^+^ T-cell responses. The vaccine is 95% effective against COVID-19; reactogenicity is generally mild or moderate. The Centers for Disease Control and Prevention (Washington, DC, USA) recently reported, however, that anaphylaxis occurs at an estimated rate of 11.1 cases per million first doses administered [76]. Additionally, BNT162b2 must be shipped and stored at −70 °C temperatures and requires two doses to be fully effective, contributing to supply chain problems especially in poor rural areas. Notably, a U.S. Food and Drug Administration press release dated 21 February 2021 indicated that BNT162b2 can be stored in undiluted form for up to two weeks at −25 to −15 °C. Like the BNT162b2 vaccine, the mRNA-1273 vaccine produced by Moderna is a LNP encapsulated nucleoside-modified mRNA-based vaccine that encodes the stabilized prefusion spike glycoprotein trimer of SARS-CoV-2 that is required for host cell attachment and viral entry [77]. mRNA-1273 was created in approximate 2 months following disclosure of the draft viral genome, a record turnaround for a vaccine candidate [78]. Vaccination induced binding and neutralizing antibody titers in a phase 1 trial that were equivalent to those found in convalescent serum samples. A spike epitope-specific CD4^+^ T-cell response, which was biased toward Th_1_-type cytokine expression, was also found; the CD8^+^ T-cell response, however, was marginal [78]. The vaccine exhibited 94.1% efficacy in preventing symptomatic infection and 100% efficacy in preventing severe COVID-19 in a phase 3 clinical trial [77]. Adverse events were mild or moderate. Unlike the BNT162b2 vaccine produced by Pfizer-BioNTech, mRNA-1273 is expected to remain stable for 30 days when stored at 2 to 8 °C (Moderna Inc. 16 November 2020 press release).

The CVnCoV vaccine produced by CureVac is a LNP formulated, mRNA-based SARS-CoV-2 vaccine that encodes the full-length spike protein stabilized in a prefusion conformation. The mRNA component was optimized for high immunogen expression and moderate activation of innate immune responses using proprietary mRNA technology that includes non-chemically modified nucleotides and a GC-enriched open reading frame. The formulation induced strong humoral and cell-mediated responses in NHP that protected from subsequent challenge with SARS-CoV-2 [79]. Antibody titers produced by vaccinated participants enrolled in a phase 1 study were comparable to patients who recovered from SARS-CoV-2 infections (ClinicalTrials.gov Identifier: NCT04449276) [80]. No serious vaccine-related adverse events occurred. Volunteers are currently being recruited for a phase 2b/3 trial to determine CVnCoV vaccine efficacy (ClinicalTrials.gov Identifier: NCT04652102). Notably, CVnCoV can be stored for 3 months at 5 °C presumably due to the absence of nucleoside modifications, which permits more tightly packed LNP and increased stability (CureVac, 12 November 2020 press release). In this regard, enhancing the thermostability of mRNA vaccines and enabling their distribution and long-term storage at higher temperatures is an immediate goal of ongoing studies. The three mRNA-based SARS-CoV-2 vaccines currently in clinical use are summarized and compared in Table 1.

The emergence of three SARS-CoV-2 variants has raised enormous concern regarding the efficacies of the currently available COVID-19 vaccines [82]. The B.1.1.7., B.1.351, and P1 variants, first identified in the United Kingdom, South Africa, and Brazil, respectively, all possess mutations that affect the spike protein and, often, the receptor-binding domain that mediates binding to ACE2 receptors expressed on human cells. These mutations promote transmission, rapid spread, and higher virus burden post-infection, but do not seem to be associated with more severe disease. Sera obtained from individuals vaccinated with mRNA-1273 (Moderna) exhibited high-titer neutralizing antibody against the B.1.1.7 and B.1.351 variants, but a reduction in neutralizing antibody specific for P1 [82]. Laboratory tests indicate that immunization with the BNT162b2 vaccine (Pfizer-BioNTech) will also be effective in preventing infection by the SARS-CoV-2 B.1.1.7 and B.1.351 variants [83]. While it is reassuring that mRNA-1273 and BNT162b2 vaccines provide protection against these two emerging SARS-CoV-2 variants, it is equally reassuring to know that the flexibility provided by mRNA-based vaccine technology enables the periodic reformulation of vaccines so that they better match circulating viral variants [84].

### 4.3. Allergies and Autoimmune Diseases

While current research efforts are focused on cancer and infectious diseases, the results of recent animal studies demonstrate the potential use of RNA vaccines to prevent or treat allergies and autoimmune diseases. Allergen-specific immunotherapy is an effective treatment for type I hypersensitivity reactions. Prophylactic intervention in young children to induce an immunological bias that prevents Th_2_ sensitization has been proposed to stop the increase in patient numbers [85]. Mice vaccinated intradermally with mRNA encoding the grass pollen allergen Phl p5 exhibited a Th_1_-type response and IFN-γ production. Vaccination suppressed Th_2_ cytokine production, IgE synthesis, and lung eosinophilia, supporting the efficacy of an RNA-based vaccine in suppressing sensitization to type I allergic reactions [86].

There is a clear need for new approaches to treat autoimmune diseases, such as type 1 diabetes and multiple sclerosis, that control autoreactive T cells while circumventing the adverse effects associated with broad-acting therapeutics and systemic suppression. Accordingly, Krienke and coworkers recently reported that an mRNA vaccine construct, which encoded disease-related autoantigens, negated all signs of experimental autoimmune encephalomyelitis in a mouse model of multiple sclerosis [87]. Treatment was characterized by a reduction in antigen-specific effector T cells and a concomitant increase in regulatory T cells. Notably, vaccination did not impair the subsequent capacity to elicit immune responses to unrelated antigens. These findings suggest that RNA based vaccines could play a valuable role treating the more than 80 estimated autoimmune diseases.

## 5. Conclusions

The speed with which mRNA-based vaccines were developed, produced on a massive scale and used clinically to confront the COVID-19 pandemic, provides proof-of-concept that RNA-based vaccines offer a promising new approach to immunizing against disease.

## Figures and Tables

**Table 1:** mRNA-based SARS-CoV-2 vaccines.

Company	Vaccine	mRNA	Immunogen	LNP (Probable Ionizable Lipid)	Dose (μg mRNA, Twice)
**Pfizer-BioNTech**	BNT162b2	modified nucleoside	prefusion stabilized spike protein	Acuitas ALC-0315 [81]	30
**Moderna**	mRNA-1273	modified nucleoside	prefusion stabilized spike protein	Lipid H [52]	100
**CureVac**	CVnCoV	unmodified	prefusion stabilized spike protein	Acuitas ALC-0315 [81]	12

## Data Availability

No new data reported.
